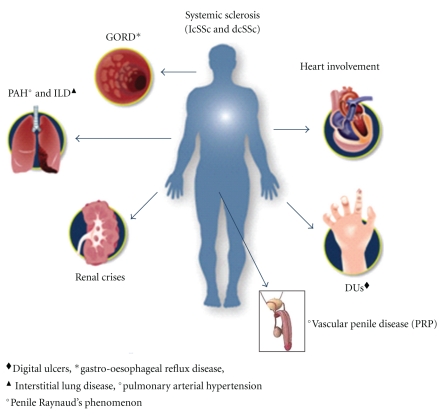# Penile involvement in Systemic Sclerosis: New Diagnostic and Therapeutic Aspects

**DOI:** 10.1155/2010/708067

**Published:** 2010-10-05

**Authors:** Antonio Aversa, Roberto Bruzziches, Davide Francomano, Edoardo Rosato, Felice Salsano, Giovanni Spera

**Affiliations:** ^1^Department of Experimental Medicine, Internal Medicine Unit, Università degli Studi di Roma ‘La Sapienza', 00161 Rome, Italy; ^2^Department of Clinical Medicine, Clinical Immunology Unit-Scleroderma Center, Università degli Studi di Roma ‘La Sapienza', 00161 Rome, Italy

## Abstract

Systemic Sclerosis (SSc) is a connective tissue disorder featuring vascular alterations and an immunological activation leading to a progressive and widespread fibrosis of several organs such as the skin, lung, gastrointestinal tract, heart, and kidney. Men with SSc are at increased risk of developing erectile dysfunction (ED) because of the evolution of early microvascular tissutal damage into corporeal fibrosis. The entity of penile vascular damage in SSc patients has been demonstrated by using Duplex ultrasonography and functional infra-red imaging and it is now clear that this is a true clinical entity invariably occurring irrespective of age and disease duration and constituting the ‘‘*sclerodermic penis*”. Once-daily phosphodiesterase type-5 (PDE5) inhibitors improve both sexual function and vascular measures of cavernous arteries by improving surrogate markers of endothelial dysfunction, that is, plasma endothelin-1 and adrenomedullin levels, which may play a potential role in preventing progression of penile fibrosis and ED. Also, the beneficial effect of long-term PDE5i add-on therapy to SSc therapy in the treatment of Raynaud's phenomenon is described.

## 1. Introduction

Systemic Sclerosis (SSc) is a connective tissue disorder featured by vascular alterations and immunological activation leading to progressive and widespread fibrosis of several organs such as skin, lung, gastrointestinal tract, heart, and kidney [1, 2]. The typical hallmark of SSc is a microvascular involvement, while macrovascular involvement is not well documented in these patients, but the majority of authors agree that its prevalence is similar to general population [3]. Vascular involvement in SSc has been believed to be limited to digital arteries [4]. It is extremely rare that SSc patients without vascular risk factors have macrovascular lesions above the elbow or knee. However, a relatively high incidence of vascular involvement between the digits and the elbow or knee has been described [5]. Early disease is mediated through microvascular dysfunction secondary to a number of factors including endothelial damage, overexpression of specific adhesion molecules, and perivascular inflammatory cell infiltration [6]. These changes make endothelium unable to carry out its functions in the regulation of vascular tone, coagulation, adhesions and migration of blood cells, transportation of nutrients, achieved through production of a complex array of molecules including vasodilators (e.g., nitric oxide: NO), vasoconstrictors (e.g., endothelin-1: ET-1), and cell adhesion molecules (e.g., selectins and integrins) [7]. The endothelial dysfunction can explain some major clinical symptoms of SSc such as Raynaud's phenomenon (RP), fingertip ulcers and gangrene, pulmonary arterial hypertension and erectile dysfunction (ED) (Figure 1). Irrespective of the classification of the disease, SSc is typically associated with RP that is characterized by microvascular damage, high plasma adrenomedullin and ET-1 levels, reduced production of NO [8–11]. 

 Similarly, at the penile level, there occurs a consistent vascular damage that almost invariably determines, as a consequence of both endothelium damage [12–16] and increased fibrogenesis [17], the so-called “*sclerodermic penis”*. In fact, the prevalence of ED in men with SSc has been reported as high as 80% [18] and it can be considered an end-organ disease involving both macro and microvascular damage.

## 2. Pathophysiology of Sclerodermic Erectile Dysfunction

Hormonal derangement is a common finding in SSc patients even if a hormonal basis for impotence, that is, abnormalities in serum testosterone, follicle-stimulating hormone, luteinizing hormone, prolactin, and oestradiol, has never been demonstrated. Neurological causes could also be excluded. In contrast, penile blood pressure, but not ankle blood pressure indices, were found to be diminished [19]. Duplex sonography measurements in male SSc patients show impaired peak systolic velocities (PSVs) in penile arteries and also the presence of veno-occlusive dysfunction. The latter is often associated with the identification of diffuse hyperechogenic “spots” inside the corpora cavernosa, along with a thickening of the tunica albuginea and is consistent with the presence of a high degree of corporeal fibrosis [20]. In a recent angiographic study it has been demonstrated that the prevalence of coronary artery disease in SSc patients is not different from a control group [21]. In accordance, our studies found that the intima-media-thickness (IMT) of the common carotid artery of patients with ED in the context of SSc is normal, thus confirming that advanced atherosclerosis occurs late in the course of disease. By contrast, endothelial dysfunction is present early, as we were able to demonstrate with impaired thermal recovery of the penis after cold exposure [22]. Taken together, these data indicate an altered arterial blood flow in the absence of general atherosclerosis as it is common in end-organ disease. Indeed, an increased collagen synthesis by smooth muscle cells and the accumulation of extracellular matrix had been already demonstrated in patients with SSc [23], and it is known that under hypoxic conditions of various origin, transforming growth factor beta (TGF*β*1), platelet-derived growth factor (PDGF) and its receptors are overexpressed in the corpora cavernosa [24]. TGF*β*1 and PDGF have both been identified as important regulators of the collagen and extracellular matrix synthesis by smooth muscle cells and also act as smooth muscle mitogens. Under hypoxic conditions, human penile smooth muscle cells also release ET-1 and induce ET-B receptor expression, processes that in turn are strongly increased by TGF*β*1 and ET-1 itself [25]. These results suggest that the molecular mechanisms by which penile hypoxia of any cause induce penile fibrosis are similar to those implicated in the fibrotic transformation of tissues in SSc patients [26]. The hypoxic and the SSc-specific processes may thus contribute to, and even perpetuate, each other in the manifestation of ED. 

 Contradictory attitudes exist about the mechanisms of the vasospastic arteriolar paroxysms—from hyperactivity of the sympathetic nervous system and “local defect” with receptor and nerve endings' dysfunctions, endothelial dysregulation and blood cells' activation to statements of primary central nervous mechanism. The venoarteriolar reflex is a local mechanism protecting the capillary bed against high hydrostatic pressure and therefore the tissue against edema. The underlying mechanism of the venoarteriolar reflex is still debated, although it is commonly believed to depend on an intact innervations of the arterioles by sympathetic vasoconstrictor fibers [27]. The modulating role of endothelium with its influence on the contractile behaviour of precapillary resistance vessels is also assumed so we can hypothesize that an autonomic nervous system dysregulation can play an important role in determining microvascular damage in sclerodermic patients. Evidence-based studies aimed to investigate neurological involvement in SSc patients are lacking.

## 3. Diagnostic Approach

The diagnosis of scleroderma is not always easy. If scleroderma is suspected, tests should be ordered to confirm the diagnosis, as well as to determine the severity of the disease. Major body changes determined by SSc are often responsible for increased morbidity and mortality, and may contribute to the occurrence of psychological disturbances such as anxiety and depression, whereas the incidence of psychotic symptoms is very low in SSc patients [28]. It is known that SSc patients are more vulnerable to depression [29], and psychological interventions including counseling with special psychotherapists and even antidepressant medication may prevent the development of depression in such patients. Further studies are needed to confirm our findings in a prospective manner if possible, and also to determine whether depression is an important prognostic indicator for quality of life in SSc. and an important breakthrough can be made in understanding the underlying mechanisms of psychiatric manifestations, in order to improve therapeutic management and quality of life. Furthermore, an accurate psychological assessment in SSc men with ED is recommended.

ED should always be investigated with appropriate questionnaires and dynamic penile Duplex ultrasound. In fact, penile vascular damage occurs in almost all SSc patients, regardless of clinical symptoms and we have suggested that investigation of these patients with Duplex ultrasound is mandatory for documenting the degree of vascular involvement since the self-administered International Index of Erectile Function questionnaire does not often match with vascular findings [20]. In addition, we have demonstrated that penile thermal proprieties of SSc patients differ from healthy controls, by using functional infrared imaging [22]. Data collected in this late pilot study demonstrate that SSc patients' penile temperature appears to be lower than that of the healthy controls. In particular, it seems that major differences are found at the level of the corpora, while minor but still significant differences are pointed out for the temperature of the glans penis. Since cutaneous temperature depends on cutaneous blood flow and thermal exchanges with deeper tissues (by convection through the arterovenous network and by conduction from vessel walls to tissues), the results seem to suggest the existence of functional alterations of both tissue properties and blood flow. Penile temperature response to thermal stress seems to confirm such a hypothesis since SSc patients counteract to and recover from cooling differently from controls. Therefore, assessing whether thermal properties and temperature control processes of the penis in SSc patients are altered could provide clues on potential ED, the progression of the illness and the effectiveness of possible treatments [22].

 After careful review of clinical and instrumental data already published in our previous papers, we suggest that there is a relationship between ED and SSc vascular damage evaluated by capillaroscopic pattern and vascular domain of Medsger Disease Severity Scale (DSS) [30]. In our opinion ED is present at onset in all patients with SSc, but early stage of ED it can be attributed to a reduced penile arterial inflow that is similar to RP of the hands and that configures the “*sclerodermic penis*”. With the progression of micromacrovascular damage in the natural course of the disease, a concomitant penile fibrosis and venocclusive dysfunction occur leading to difficult-to-treat ED. 

 For these reasons, a multidisciplinary approach to SSc patients would involve the rheumatologist, the uro/andrologist and the psychiatrist to comprehensively evaluate all diagnostic aspects.

## 4. Treatment

The treatment of ED in SSc suggest to modify or revert risk factors for ED, including lifestyle, psychological or drug-related factors, but such treatments are often unsatisfactory and limited by frequent side effects in the long-term. Three different phosphodiesterase type-5 (PDE5) inhibitors are currently available: sildenafil, vardenafil and tadalafil. Several randomized trials have demonstrated the efficacy of this class of medications, but there are no compelling data to support the superiority of one PDE5 inhibitor over another in non-SSc patients. The three PDE5 inhibitors share many pharmacological and clinical characteristics. Initial studies involving animal models, data from open-label, uncontrolled trials involving patients with pulmonary arterial hypertension, and a small randomized, controlled studies involving patients with idiopathic pulmonary arterial hypertension suggest that PDE5 inhibitors are beneficial in the treatment of pulmonary arterial hypertension [31–33]. Recently some authors have shown that sildenafil may be complimentary in the treatment of RP refractory to conventional drugs [34, 35]. Several studies have corroborated the efficacy of PDE5 inhibitors in the treatment of ED related to several internistic disease such as atherosclerosis, mellitus diabetes, arterial hypertension [36]. This improvement is confirmed also by sexual questionnaires and suggests PDE5 inhibitors as an adjunctive therapy that might possibly reverse the process of endothelial damage leading to vascular disease in SSc. In a recent study, we demonstrated that once-daily tadalafil is able to decrease the mean number of Raynaud's attacks by reducing surrogate markers of vascular damage such as adrenomedullin and ET-1 plasma levels. The results of that study lead us to postulate the beneficial effect of adding daily long-term PDE5 inhibitors to the medical treatment of SSc [37]. The use of endothelin-receptor antagonists (ERAs) in SSc is actually deserved to patients with severe pulmonary arterial hypertension. The possibility of an add-on effect of ERAs to PDE5i is to be considered in future studies. At the moment, no proof-of-fact that the use of ERAs alone may improve ED is present.

 In the era of PDE5 inhibitors, we believe that the implantation of a penile prosthesis may be considered only in patients who fail pharmacotherapy or who prefer a permanent solution of their problem. The choice of prosthesis is depends from patients' preference and his manual dexterity. This solution may have an high satisfaction rate as in the non-SSc population but must take into account that it is not reversible. Finally, mechanical failures and infection has the same occurrence (1%–3%) than the general population [38].

## 5. Conclusion

Long-standing SSc is almost always associated with the presence of some degree of ED, because of micro and macrovascular disseminated damage through cavernosal penile arteries and capillaries and subsequent cavernosal fibrosis. As a consequence, ED should be systematically questioned and counselled in any patient presenting with SSc by a specialized team. Although robust data on treatment options for ED in the SSc population are not yet available, empirical treatment should be started with a daily or alternate day regimen of a long-acting PDE5i after addressing modifiable risk factors for ED. Second-line treatment decisions require the cooperation between expert specialists in order to maximize medical therapy benefits for underlying conditions.

## Figures and Tables

**Figure 1:**